# Spatio-temporal modelling of routine health facility data for malaria risk micro-stratification in mainland Tanzania

**DOI:** 10.1038/s41598-023-37669-x

**Published:** 2023-06-30

**Authors:** Sumaiyya G. Thawer, Monica Golumbeanu, Samwel Lazaro, Frank Chacky, Khalifa Munisi, Sijenunu Aaron, Fabrizio Molteni, Christian Lengeler, Emilie Pothin, Robert W. Snow, Victor A. Alegana

**Affiliations:** 1 Swiss Tropical and Public Health Institute, Allschwil, Switzerland; 2 University of Basel, Basel, Switzerland; 3 Ministry of Health, Dodoma, Tanzania; 4 National Malaria Control Programme, Dodoma, Tanzania; 5 Clinton Health Access Initiative, New York, USA; 6 Population Health Unit, KEMRI-Welcome Trust Research Programme, Nairobi, Kenya; 7 Centre for Tropical Medicine and Global Health, Nuffield Department of Clinical Medicine, University of Oxford, Oxford, UK; 8 World Health Organization, Regional Office for Africa, Brazzaville, Republic of Congo

**Keywords:** Malaria, Statistics

## Abstract

As malaria transmission declines, the need to monitor the heterogeneity of malaria risk at finer scales becomes critical to guide community-based targeted interventions. Although routine health facility (HF) data can provide epidemiological evidence at high spatial and temporal resolution, its incomplete nature of information can result in lower administrative units without empirical data. To overcome geographic sparsity of data and its representativeness, geo-spatial models can leverage routine information to predict risk in un-represented areas as well as estimate uncertainty of predictions. Here, a Bayesian spatio-temporal model was applied on malaria test positivity rate (TPR) data for the period 2017–2019 to predict risks at the ward level, the lowest decision-making unit in mainland Tanzania. To quantify the associated uncertainty, the probability of malaria TPR exceeding programmatic threshold was estimated. Results showed a marked spatial heterogeneity in malaria TPR across wards. 17.7 million people resided in areas where malaria TPR was high (≥ 30; 90% certainty) in the North-West and South-East parts of Tanzania. Approximately 11.7 million people lived in areas where malaria TPR was very low (< 5%; 90% certainty). HF data can be used to identify different epidemiological strata and guide malaria interventions at micro-planning units in Tanzania. These data, however, are imperfect in many settings in Africa and often require application of geo-spatial modelling techniques for estimation.

## Introduction

The importance of targeting interventions through adequate malaria planning and informed decision making has been emphasized by the recently launched World Health Organization (WHO) High Burden High Impact initiative (HBHI)^1^. This initiative encourages national malaria control programs (NMCPs) across Africa to use local, routine and survey data to stratify malaria risk at the national and sub-national levels and accordingly define appropriate targets for their malaria strategic plans^1^. To date, national stratification using available routine data from health information systems has been conducted in several African countries including Burkina Faso^2^, Eritrea^3^, Ghana^4,5^, Kenya^6,7^, Madagascar^8–10^, Malawi^11^, Mali^12^, Namibia^13,14^, Rwanda^15^, South Africa^16^, Swaziland^17^, Tanzania^18,19^, Uganda^20^, Zambia^21,22^ and Zimbabwe^23^ with most utilizing incidence as a metric of malaria measure. The sources of data used by NMCPs for national stratification vary between countries and is dependent on the availability, access and quality of information^24,25^.

In recent years, the launch of the WHO test and treat policy^26^ along with investments to digitize the health management information system (HMIS) under the electronic district health information software (DHIS2) has resulted in gradual improvements in the quality and completeness of routine data from health facilities (HFs). Routine data offers a source of data that is temporally and spatially much more comprehensive than parasite prevalence from periodic household surveys. They provide real-time and spatially granular information which is essential for effective monitoring and timely planning of interventions.

Most NMCPs in many countries have some form of stratified maps of malaria risk based on aggregating routine data, climatic stratification, or parasite prevalence^27,28^. These stratification maps are usually produced at the higher administrative levels (macro)—or lower administrative levels (micro). Recent malaria guidelines advocate for the use of routine data for monitoring and evaluation at country levels and demonstrate its utility as part of donor requests for monitoring progress^29^. However, at the micro-planning units, limitations of routine HF data including its availability and geographic and temporal representativeness, can limit its utility. These factors contribute to uncertainty in estimates generated from these data and has over the years hindered its direct use for decision making. For example, at the micro-levels, not all areas have HFs resulting in long commuting distance for communities to reach the nearest HF. Thus, the estimation of disease indicators for these communities is not straight forward without application of appropriate spatial modelling techniques. Routine data from communities in areas with HFs may have additional deficiencies such as reporting completeness^30^. Conducting disease specific micro-stratification is important for understanding heterogeneity of disease risk. The ability to stratify malaria risk at a finer level will lead to even better spatially targeted responses aligned to the HBHI concept. This becomes increasingly beneficial in areas moving towards lower transmission risk to quantify the levels of heterogeneity and support elimination efforts.

For empirical routine data to provide accurate malaria estimates, all community fever cases should ideally reach HFs, be tested and accurately captured within the DHIS2^24^. However, this is often not the case. Routine data do not account for factors such as treatment seeking rates, health utilization behaviors, the underlying heterogeneous distribution of the population and the differing testing rates between transmission settings. All of these, can potentially under/over-estimate malaria risk^16,24^. In the absence of complete and perfect empirical data, statistical modelling techniques represents a practical way to close these gaps and obtain best estimates for all settings. Spatio-temporal models have been extensively used for various diseases^24,31–33^ and are based on the principles that data are spatially correlated and observations in adjacent areas will be more similar than observations that are farther away, thereby smoothing risk in space and time according to a neighborhood structure^34^. The models allow to efficiently handle incomplete or missing data, account for potential biases^21,24,35^ and are also useful for understanding the associated levels of uncertainty in the data.

Mainland Tanzania has formally adopted macro-stratification as part of its National Malaria Strategic Plan (NMSP) 2021–2025^36^ aimed at providing tailored combinations of interventions according to council level epidemiological risk^18,19,36^. Multiple metrics have been previously used to provide a simplified risk-strata per council based on survey data from school children^38^ and routine data from DHIS2^18^. To further account for the intra-council heterogeneity and support decentralized planning, the stratification was extended to the ward level to develop a micro-stratification risk map using aggregated routine data as highlighted in previously published work^36,37^. The routine metrics utilized in this micro-stratification approach^37^ included annual parasite incidence (API), test positivity rate (TPR) confirmed with malaria Rapid Diagnostic Test (mRDT), and test positivity rates from antenatal care clinics (ANC TPR). Furthermore, inclusion of data was limited to HFs with a minimum of 50% completeness of reporting. The use of empirical routine data in this micro-stratification approach however, did not adjust for the existing spatial and temporal gaps nor the related uncertainties, thereby resulting in an incomplete ward-level stratification where 5% of all the wards had no HFs and thus no stratification could be conducted here^37^.

Here, we used Bayesian conditional auto-regressive (CAR) spatio-temporal modelling techniques to leverage all the available routine data collected over 36 months from all reporting HFs across wards in mainland Tanzania. The aim was to improve previous micro-stratification efforts in mainland Tanzania^37^. In this study, we focused on the mRDT TPR, a widely used malaria metric reported by routine health systems^6,39–49^. Malaria TPR has been shown to be significantly associated with malaria incidence and a strong predictor of malaria transmission^43–45^. It offers a more consistent and acceptable case definition since it provides a clearer denominator and does not require information on HF catchment population that remains largely undefined^44,50^.

## Results

### Routine data coverage and description

A total of 7878 HFs offering laboratory services and performing testing with mRDTs were included in the analysis for the reporting period 2017–2019 (Table 1). During this period, a total of 228,717 facility monthly reports were received resulting in an overall reporting rate of 80.7% across 93.7% wards. Dispensary, laboratories and clinics represented most of all the HFs (85.7%), followed by health centers (10.8%) and hospitals (3.5%) (Supplementary Fig. S1). Of the total malaria tests performed by mRDT (n = 56,546,468) in the period of analysis, 15,454,915 (27.3%) were positive for malaria, showing a marked variation in the crude malaria TPR from 0.0 to 82.5% across all wards. The number of HFs per ward widely ranged with higher number of HFs found in urban wards compared to rural wards. A large number of wards consisted of only one (27.9%) or two (29.4%) HFs. On the whole, 6.3% of wards had no HFs or non-reporting HFs, corresponding to 4% of the total population.

**Table 1:** The coverage and completeness of malaria Test Positivity Rates (TPR) across wards in mainland Tanzania from 2017 to 2019.

# of Health Facilities Performing mRDT Testing	# of Wards	% of Wards			Population Residing (%)	Facility Reporting Rates (%)	mRDT Confirmed Malaria Cases	Total Tested with mRDT	Average TPR (%) (Min–Max)
		Urban	Mixed	Rural					
0	208	35.1	12.0	52.9	2,094,992 (4%)	-	-	-	-
1	924	12.2	7.7	80.1	10,887,759 (20%)	86.1	2,906,562	9,019,681	32.2 (0.0–82.5)
2	974	7.5	7.8	84.7	13,249,794 (25%)	84.9	4,997,646	14,366,228	34.8 (0.0–79.9)
3	594	11.8	14.0	74.2	9,534,633 (18%)	83.2	3,379,948	10,604,367	31.9 (0.3–81.3)
4	287	15.7	17.8	66.6	5,987,459 (11%)	80.1	2,057,873	7,126,957	28.9 (0.6–71.1)
5	155	28.4	21.9	49.7	3,730,785 (7%)	76.7	1,005,588	4,925,657	20.4 (0.7–63.6)
6	64	31.3	18.8	50.0	1,797,811 (3%)	73.6	433,992	2,280,514	19.0 (0.8–69.9)
7	40	65.0	20.0	15.0	2,094,947 (4%)	70.2	165,987	2,206,765	7.5 (0.7–52.1)
8	27	66.7	18.5	14.8	1,426,109 (3%)	71.1	178,763	1,838,587	9.7 (0.6–43.3)
9	12	66.7	25.0	8.3	614,121 (1%)	64.1	163,956	979,849	16.7 (3.7–51.5)
10 +	26	92.3	7.7	0.0	2,301,807 (4%)	65.7	164,600	3,197,863	5.1 (0.7–28.2)
7878	3311	15.5	11.2	73.3	53,720,216	80.6	15,454,915	56,546,468	27.3 (0.0–82.5)

### Model selection

Assessment of the coefficients of the predictors selected from the covariates selection procedure (Supplementary Fig. S3) showed that Enhanced Vegetation Index (EVI) (Coefficient: 0.078; Standard Error: 0.002), Night Time Lights (NTL) (− 0.043; 0.002) and Temperature Suitability Index (TSI) (0.150; 0.004) were significant predictors of malaria TPR and were therefore included in the analysis.

Comparison of the Deviance Information Criteria (DIC) values between the three model specifications showed that model C had the lowest DIC value (304,069.5) when compared to model A (306,978.1) and model B (307,065.9) (Supplementary Table S1). Improving the model complexity improved the model goodness of fit and thereby Model C was selected and implemented. Model validation statistics were computed to validate the model performance and are summarized in Supplementary Table S1. The MAE of the selected Model C was computed to be 0.04 suggesting good model precision, the RMSE was 0.06 suggesting low bias and the R2 was 0.91 suggesting a good predictive performance of the model.

Table 2 presents the posterior parameters for the selected model C. EVI (Posterior mean; confidence interval—0.236; 0.231–0.241) and TSI (0.579; 0.511–0.647) were positively associated with malaria TPR indicating that vegetation index and temperatures are favorable for increasing the risk of transmission. As expected, NTL (− 0.300; − 0.371 to − 0.229) showed a negative correlation to the malaria risk implying areas in rural settings are more prone to malaria risk. All the model parameters were significant at the 95% credible interval.

**Table 2:** Posterior model parameter estimates.

Parameter	Posterior Mean (95% CI) (Log odds scale)
Intercept	–1.594 (–1.692 to –1.495)
EVI	0.236 (0.231–0.241)
NTL	–0.300 (–0.371 to –0.229)
TSI	0.579 (0.511–0.647)

### Heterogeneity of predicted malaria TPR at ward level

**Figure 1:**
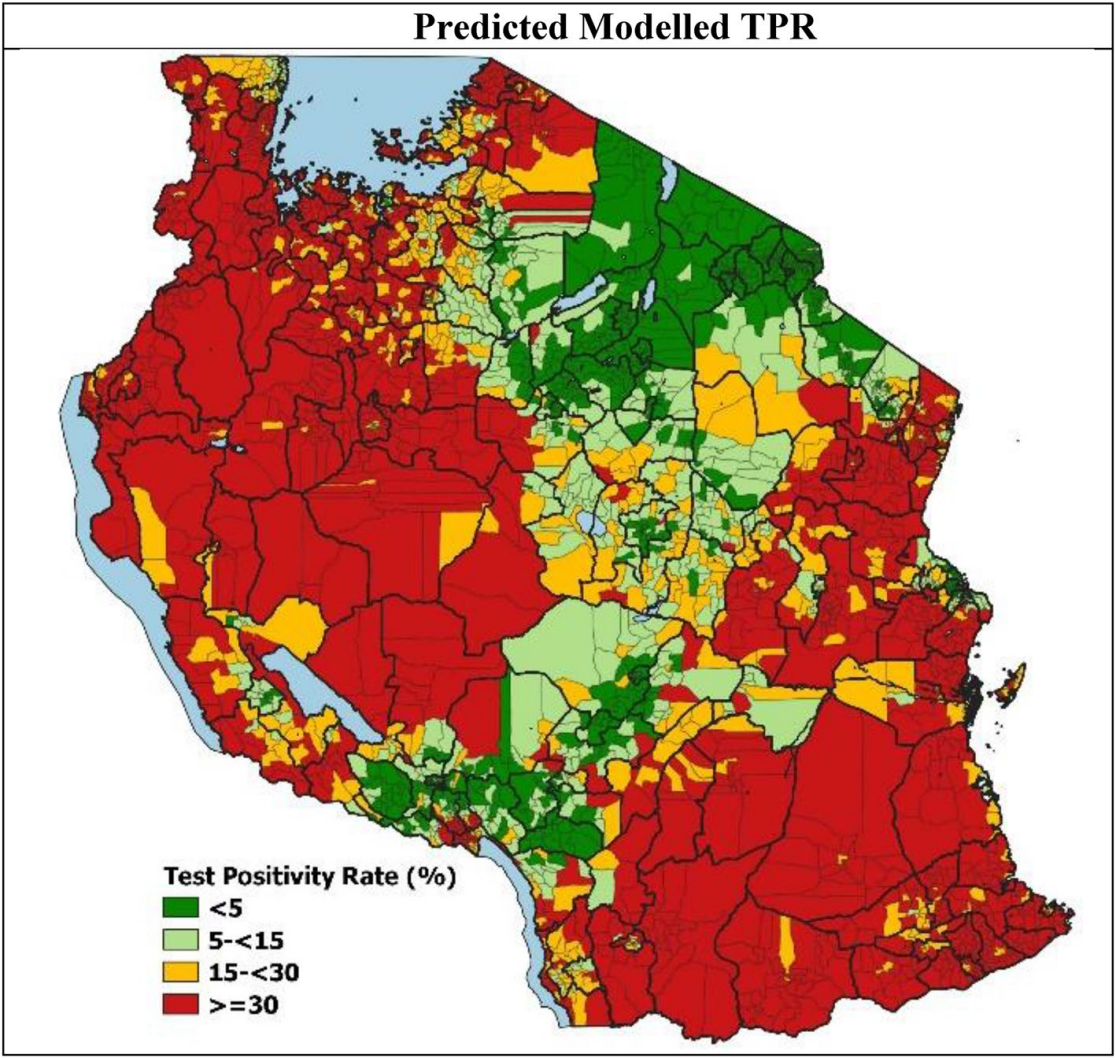
Predicted malaria Test Positivity Rates (TPR) in mainland Tanzania.

The heterogeneity in the final modelled malaria TPR risk (Fig. 1) is evident across the country with higher transmission levels seen in the North-West and South-East parts of the country, whilst lower transmission levels are seen in the central corridor running from the North-East to South-West parts of the country. At the national level, the predicted mean malaria TPR for the period of analysis was 25.6% (95% credible interval 23.9–27.6) with heterogeneity across the wards ranging from 0.2% (0.1–0.4) to 81.4% (80.9–81.9%).

Following classification of the estimated malaria TPR values into risk strata using the NMCP defined thresholds (Supplementary Table S2), 1348 (40.7%) wards were assigned to high transmission risk strata, 583 (17.6%) wards to moderate transmission, 633 (19.1%) wards to low transmission, whilst 747 (22.6%) wards to the very low transmission strata. The average estimated malaria TPR distribution per risk stratum is summarized in Table 3.

**Table 3:** Distribution of wards by transmission strata.

Malaria TPR Risk Strata	# of Wards (%)	# of Population Residing (%)	Average Predicted Malaria TPR (Credible Interval %)
Very Low (< 5%)	747 (22.6%)	13,795,566 (25.7%)	2.5 (1.9–3.3)
Low (5- < 15%)	633 (19.1%)	11,967,597 (22.3%)	9.1 (8.0–10.7)
Moderate (15- < 30%)	583 (17.6%)	8,894,349 (16.6%)	22.2 (20.5–24.5)
High (≥ 30%)	1348 (40.7%)	19,062,704 (35.5%)	47.5 (44.9–50.4)
	3311 (100%)	53,720,216 (100%)	25.6 (23.9–27.6)

### Interpreting uncertainty in malaria TPR at the ward level

The model exceedance and non-exceedance probabilities provided some level of confidence in the assigned risk strata to allow NMCPs and the council health teams to efficiently plan targeted interventions at the micro levels. This is particularly useful in the extreme high and very low transmission risk areas where the largest transition in intervention packages from control to elimination strategies are observed^36^.

**Figure 2:**
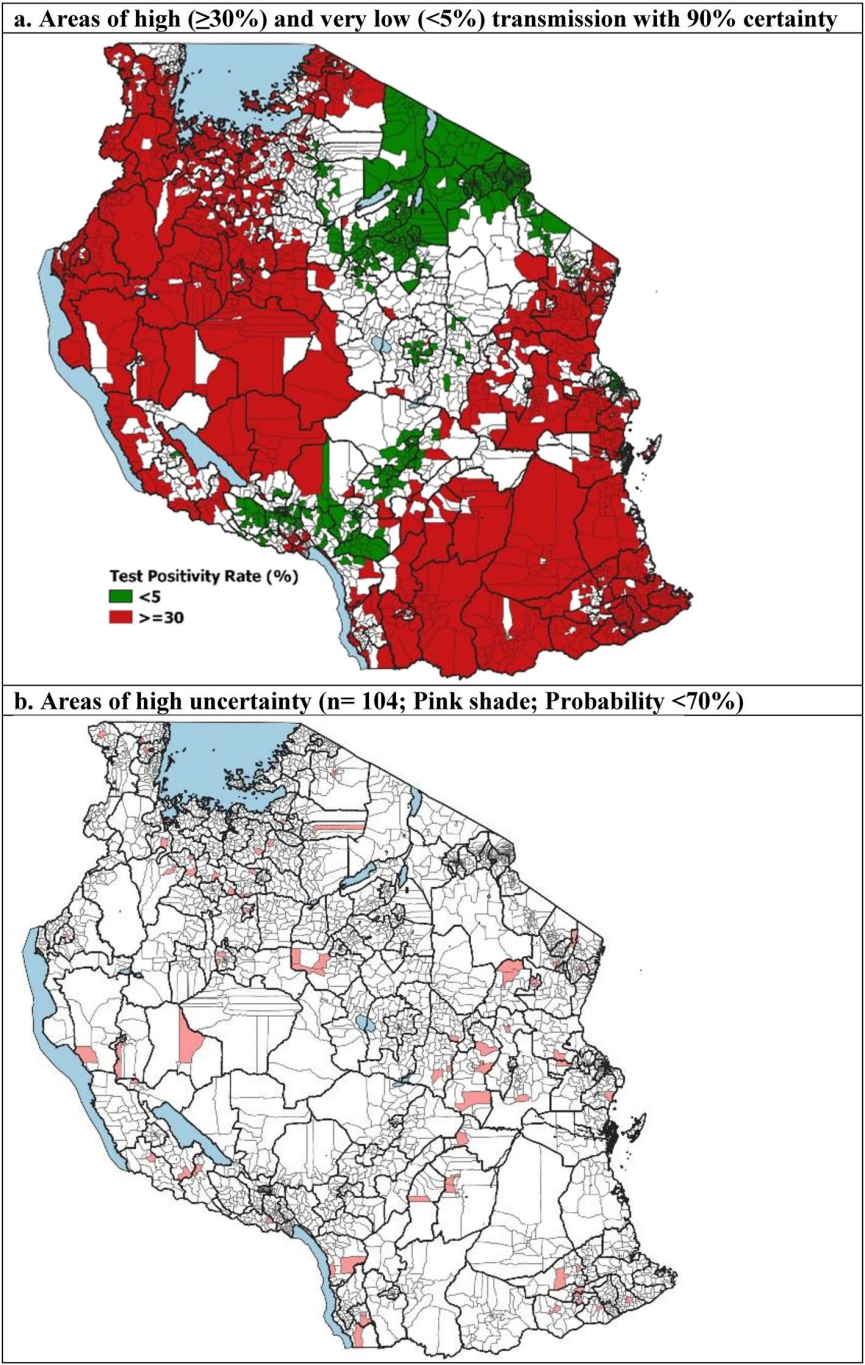
Exceedance and non-exceedance probability of predicted malaria Test Positivity Rates (TPR).

A malaria TPR of ≥ 30% is the threshold set by the NMCP to denote areas with high transmission and that qualify for the most intensive control interventions to reduce transmission. Approximately 17.7 million people (33%) were estimated to reside in 1227 wards with a probability of ≥ 90% which shows a high transmission risk. The majority of this population was located predominantly in the North-West and South-East of the country. Another 11.7 million people (22%) resided in 662 wards with very low transmission risk of < 5% and were found largely in the North-East councils (Fig. 2a). These indicate areas where elimination strategies such as strengthening surveillance systems should be considered^36^. Approximatively 1.2 million people resided in 104 wards where the assigned risk strata had large levels of uncertainty (probability < 70%) (Fig. 2b).

Comparison of the risk strata estimated from the model with the empirical estimates of malaria TPR (which did not account for uncertainty) showed 7.4% of the total wards to be misclassified. Amongst these, 68 wards (2.2%) in the low strata were found to be misclassified to the very low risk strata by the empirical malaria TPR. Another 32 wards (1.0%) in the high risk strata were found to be misclassified to the moderate risk strata. These represent areas where the largest impact of misclassification would likely be observed due to the significant differences in the intervention strategies in these strata.

## Discussion

In this work, a Bayesian spatio-temporal modelling framework was used to leverage routine information from HFs and provide robust estimates of malaria risk at ward level. The model allowed to smoothen the risk and fill the spatial and temporal gaps in routine data, handle the associated uncertainty in a robust manner and account for any spatial and temporal dependencies in the data. The analysis highlighted the sub-council level spatial heterogeneities in malaria TPR with higher transmission particularly seen in the North-West and South-East parts of the country. These areas have traditionally been shown to have similar patterns of higher prevalence^18,38,51–53^. Factors potentially contributing to resilience in changes to the risk could be due to the geographic location, climatic factors and socio-economic factors amongst many.

As countries begin to transition towards lower malaria transmission, the need to monitor the increasing heterogeneities at finer scales and inform appropriate tailored strategies becomes critical. HF data represents an essential source of local information describing the dynamics of the malaria situation with a high level of resolution in time and space. Understanding their structure and representativeness can be useful to replace modelled prevalence estimates derived from sparse cross-sectional surveys that is widely considered as the current gold standard. Nevertheless, at the local administrative levels, incomplete HF reporting or non-reporting HFs create varying degrees of spatial and temporal data gaps. Moreover, as observed in this analysis, about 57.3% wards had only 1 or 2 reporting HFs, thereby contributing to a higher level of uncertainty.

The modelling framework used here allowed for a more robust estimation of malaria TPRs by borrowing information from neighboring wards, rather than relying only on limited information from one single ward. In addition to adjusting for the missing information, the approach provides measures of uncertainty that are required to make relevant policy decisions. Previous work done in mainland Tanzania^36,37^ used combinations of empirical routine data to develop a micro-stratification risk map, but that approach did not consider the uncertainty in transmission risk for the population at risk. This is important to allow NMCPs to understand the fidelity of estimates, understand progress made towards achieved targets and more confidently transition malaria strategies. The current paper builds on this by providing a more robust estimate of risk. By presenting the risk in terms of exceedance and non-exceedance probabilities, the developed model allows programs to also identify areas with high uncertainty in their assigned risk (Probability < 70%). These areas are likely within wards in which there is a natural level of heterogeneity such as major altitudinal changes, natural swamps or man-made agricultural areas. Importantly, these would need to be differentiated from wards with poor HF reporting performances, or those with small numbers of patients tested at a HF resulting in larger uncertainty in actual estimates.

The current approach taken in this paper may be applied to other sub-Saharan African (SSA) countries that are facing challenges with incomplete and missing routine information at the higher spatial scales. In such places, particularly those moving towards lower transmission of risk, the use of real-time routine information becomes important to allow continuous analysis of the existing local heterogeneity. Using statistical models can be valuable to address some of these existing data issues. Nevertheless, continued efforts to strengthen routine surveillance systems must remain a country priority to help guide local evidence-based planning and implementation.

This study has some limitations. The approach uses routine data that are only representative of the population who seeks care and are laboratory tested. It therefore does not capture the variations in testing rates, infections within the communities that do not reach the facility, or those that are asymptomatic. The unavailability of treatment seeking information at ward level limited the analysis to account for this important factor. Using a combination of metrics from both routine and survey sources could further improve the estimates. Future work may look into leveraging information from both sources to better understand the relationship between the data sources and how well they reflect the different components of the transmission system. Establishing this relationship would also be important to better develop thresholds used for defining risk categories. To date, cut-offs used for defining malaria risk are mainly based on pragmatic, plausible criteria but not linked to likely biological/ epidemiological impacts of specific interventions. There is also a need to consider other layers of malaria-related information to further increase the value of malaria TPR for decision making and provide a more holistic approach to inform malaria policies sub-nationally.

The CAR modelling approach used aggregated estimates per ward and thereby assumed the ward administrative boundaries to represent the catchment population for HFs within wards. This can have several implications. Firstly, it did not account for differing facility utilization behaviors and population movements across neighboring ward borders. Many factors can drive patient choices such as the size of HFs, distance, perceptions and costs^24^. Using geo-statistical methods to account for the geo-spatial location of HFs as well as incorporating information on behaviors driving facility usage can better inform the risk estimates. Secondly, the use of aggregated data can mask underlying data quality issues thereby limiting the understanding of the true nature of data^54,55^. Finally, the use of aggregated data poses the challenge of the modifiable areal unit problem (MAUP) which is a common geographical statistical problem. This occurs when results are affected by variability introduced through aggregating data or due to changes in the polygon shape used in the analysis^56^. In this work, data were aggregated to the ward level for providing estimates at a resolution that is programmatically meaningful for micro-stratification.

The use of the complex analytical methodologies for dealing with incomplete data demands analytical skills largely beyond the capacity of most NMCPs. Hence, it is important that such methods remain within local research institutions with the required know-how for annual monitoring. Increased usage of maps for local decision making by NMCPs was recently shown to be associated with factors such as knowledge and understanding of the data sources and their limitations, and also trust and perceived ownership of the data^28^. Therefore, capacitating NMCPs to establish a high-quality surveillance system and to interpret the data after an appropriate analytical process represents a sustainable way of promoting data use for decision making^24^.

## Conclusion

This work demonstrated the potential of routine HF data to identify different epidemiological strata and thereby providing the malaria program with an evidence base to guide malaria interventions at micro-planning units in Tanzania. These data, however, are imperfect in many settings in Africa and often require application of geo-spatial modelling techniques for estimation. These techniques allow for filling the existing spatial and temporal data gaps, accounting for statistical uncertainty, and leveraging this rich source of information for optimizing micro-planning of interventions.

## Methods

### Geographical scope and context

Mainland Tanzania is organized into multiple administrative levels. The country has 26 administrative regions, divided into 184 councils. The councils represent the main administrative level responsible for resource allocation and tailoring interventions as per the national guidelines. Councils are further divided into wards, which serve as the lowest resources allocation and disease reporting unit. A total of 3311 wards have been defined according to the 2012 national census for mainland Tanzania (Supplementary Fig. S6). There is a range from 2 to 43 wards per council, depending on the size of the council, altitudinal variation and population density.

### Routine health facility data processing

Data from 7878 (93%) reporting HFs across 3103 (93.7%) wards in mainland Tanzania were used to assemble malaria TPR data (Supplementary Fig. S1). The remaining wards (6.3%) did not have reporting HFs. Aggregated routine data (see data aggregation description below) from the laboratory register representing all ages were obtained from HMIS/DHIS2 for 36 months (2017–2019). DHIS2 is an open source, web-based software platform for reporting, analysis, and dissemination of health data. It captures information from both the private (26%) and public (74%) HFs, and can be accessed by officials working in the health sector through registered credentials. Each month, HFs provide paper-based monthly summary reports with data that are entered into DHIS2.

Monthly laboratory testing reporting tools were introduced in HFs in October 2015 to capture: (1) the total number of malaria tests performed by blood slides and mRDT across all age groups, and (2) the number of positive malaria cases. The reporting rates have gradually improved from 49.6% in 2016 to 87.7% in 2019. mRDTs were introduced in mainland Tanzania in 2009 in several rolled-out phases before country wide scale up was achieved in 2013^57^. Currently, mRDTs are the most widely-used diagnostic method for malaria (88% of total tests performed), with only a small proportion of facilities, mainly private facilities, still using microscopy.

The indicators extracted were used to compute the mRDT TPR, defined as the proportion of the number of malaria laboratory confirmed cases (numerator) amongst the total number of mRDTs performed (denominator)..

#### Data cleaning and geocoding

In this analysis, the HMIS data consisted of monthly laboratory reports of all patients tested with mRDT and reported by all public and private HFs with available geo-coordinates. These facilities represented 92.7% (N = 7878) of all HFs offering laboratory testing and those captured in the DHIS2. The remaining 7.3% HFs did not submit any monthly laboratory reports across the entire period of analysis and were therefore excluded. No information was available on whether they simply did not report, or whether they did not test. In Tanzania, only HFs offering laboratory testing services are expected to submit the monthly laboratory reports. However, this information is not clearly demarcated in the current master HF list and therefore understanding the exact proportion of HFs that were missing in the DHIS2 was not possible.

All reports were first checked for duplicate submissions for the same month by the same HF and duplicates were removed. As the DHIS2 database in Tanzania is unable to record zero values, these are marked blank. Hence, to distinguish zero values from missing values, it was assumed that missing values of otherwise complete reports were true zeros. To ensure the correct allocation of HFs to their respective wards, the geographical coordinates of the reporting HFs were obtained from the master registry HF list of Tanzania^58^ and linked to the DHIS2 data using the unique HF identifier code. The national ward shapefile was then used to allocate the HFs to their respective wards (Supplementary Fig. S1).

#### Data aggregation and classification

The HMIS monthly data were aggregated for the whole year in order to align with the NMSP development which has cycles of three years, and we therefore provided average risk estimates for the period 2017–2019. This resulted in a total of 9214 space–time data points that were included in the analysis.

The classification of routine metrics into malaria risk categories has been previously defined in the country using prevalence survey data from school children as a gold standard. This classification was guided by a set of criteria ensuring the minimization of misallocation of councils belonging to the higher strata to the lower strata, which would have led to the largest changes in the optimal intervention packages^36^ (Supplementary Table S2). We classified the estimated malaria TPR values into risk strata using the national criteria of risk as follows: < 5% as very low transmission; 5– < 15% as low transmission, 15– < 30% as moderate transmission and ≥ 30% as high transmission (Supplementary Table S2).

### Environmental and ecological covariates

A set of biologically plausible covariates known to affect malaria risks were considered for the geo-spatial modelling^34,59^. The data were extracted from open source remote sensing platforms. The covariates included precipitation^60^, EVI^61^, TSI^62^, NTL^63^ water vapor^64^ and the average HF reporting rates within a ward (Supplementary Text S1). The covariates were standardized using the observed mean and standard deviation.

A covariate selection procedure was performed in order to select a parsimonious minimal set of covariates^59,65^. The malaria TPR data series were matched to the covariates and a non-spatial generalized linear regression model was applied using the *bestglm* package in R^66^. This approach selected the best combination of the covariates based on the lowest value of the Bayesian Information Criteria (BIC). TSI, NTL and EVI were among the selected covariates as predictors (Supplementary Text S1).

### Model specification

A Bayesian Besag-York-Mollié 2 Model (BYM2)^67^ was used to model the spatial and temporal distribution of malaria TPR at the ward level adjusting for the selected covariates. The model combined the data and prior knowledge to produce posterior probability distributions and predict smoothed malaria TPR estimates thereby filling the missing values for wards with no facility data. The model was used to estimate malaria TPR at the administrative level of the ward and accounted for prediction uncertainty across wards with incomplete data or no reporting facilities (Supplementary Text S2).

Let $y(j,k)$ represent total number of positive malaria cases at the ward $j$, $\left(j=1,\dots ,n\right)$ in year $\left(k=1,\dots ,K\right)$, and $N(j,k)$ the total people tested for malaria at ward j in year k. The malaria test positivity rate (TPR) given the selected covariates was modelled using a binomial likelihood:

$$y\left(j, k\right)|\eta \left(j,k\right)\sim Binomial\left(N\left(j,k\right),P\left(j,k\right)\right)$$

$$\eta \left(j,k\right)=logit\left(P\left(j,k\right)\right)$$

where the link with the chosen environmental and ecological covariates is made through a regression model based on a linear predictor defined as:

$$logit\left(P\left(j,k\right)\right)={\beta }_{0}+X\left(j,k\right){^{\prime}}\beta +{{u}_{j}+{v}_{j}+\gamma }_{k}$$

with ${\beta }_{0}$ the intercept, $X(j,k)$ is a set of selected covariates; $\beta$ are the corresponding regression parameters; ${u}_{j}$ corresponds to the CAR structured spatial random effect that smoothens the data according to a neighbourhood structure. The CAR model was applied to a symmetric spatial neighborhood matrix structure $W$, developed at the ward level. $W=\{{w}_{\left(h,i\right)}\}$ defines a neighborhood structure across all the wards of the country (Supplementary Fig. S4), where each element ${w}_{hi}$ connects the wards $h$ and $i$, i.e., ${w}_{hi}$ = 1 if wards share a common boundary and ${w}_{hi}$ = 0 otherwise; ${v}_{j}$ corresponds to the unstructured exchangeable component using independent and identically distributed (i.i.d) random effect and ${\gamma }_{k}$ is the temporal random effect specified using i.i.d zero-mean normally distributed random effect.

In order to test the goodness of fit, CAR models with different specifications of the spatio-temporal structures were implemented (Supplementary Table S1). Model A did not have a spatial random effect component, model B had a spatial random effect component and model C was run with a spatial and temporal random effect structure (Supplementary Table S1). The model goodness of fit was evaluated using the DIC and the best model was selected and used for subsequent analyses. The model was estimated using Integrated Nested Laplace Approximation (INLA)^68–70^ (Supplementary Text S2).

Exceedance probability (EP) and non-exceedance probabilities (NEP) calculated using the fitted spatio-temporal model (Supplementary Text S2) were used to quantify the likelihood of the malaria TPR estimates to be above the high (≥ 30%) or below the very low (< 5%) malaria risk thresholds. These thresholds represent the pre-defined, policy-relevant thresholds defined by the NMCP in Tanzania. Estimates obtained from the resulting model are only programmatically useful when NMCPs are able to interpret it with its underlying level of uncertainty^6,71^.

### Model validation

To evaluate the predictive performance of the model, a subset of 10% of the dataset was held out randomly. The predictive performance of the model was estimated by computing validation statistics on the hold out data set. The mean absolute error (MAE) was computed as a measure of the absolute differences between the observed and predicted values. The root mean square error (RMSE) was computed to provide a measure of the accuracy of the individual predictions whilst the R-squared (R^2^) was computed to provide a measure of the proportion of variation accounted for by the model (Supplementary information Text S2).

### Estimating population at risk by strata

The population for each ward was obtained from the publicly available 2012 population and housing census in Tanzania conducted by the National Bureau of Statistics^72^. Annual growth rates at the council level^73^ were applied to the ward population data to project each ward population to the period of analysis (2017–2019). These were then used to estimate the total populations residing in each of the identified malaria risk strata.

R Studio^74^ was used for performing analysis of the data downloaded from DHIS2. All maps were produced using the QGIS software version 3.4.14^75^.

### Ethics approval and consent to participate

This work utilizes secondary aggregated data for analysis for which no ethics approval was required.

## Supplementary Information

Supplementary Information.

## Data Availability

Data from routine HMIS/DHIS2 are not publicly available and were obtained by request from the NMCP of mainland Tanzania. Restrictions apply to the availability of these data and permission can be obtained with reasonable request from the Ministry of Health of mainland Tanzania.

## Abbreviations

ANC: Antenatal care
API: Annual parasite incidence
BYM2: Besag-York-Mollié 2
CAR: Conditional autoregressive
DHIS2: District Health Information Software
EP: Exceedance probability
EVI: Enhanced Vegetation Index
HBHI: High burden to high impact
HF: Health facility
HMIS: Health Management Information System
INLA: Integrated Nested Laplace Approximation
MAE: Mean absolute error
MoH: Ministry of Health
mRDT: Malaria rapid diagnostic testing
NBS: National Bureau of Statistics
NEP: Non-exceedance probability
NMCP: National Malaria Control Programme
NMSP: National Malaria Strategic Plan
NTL: Night time lights
RMSE: Root mean square error
R^2^: R-squared
TPR: Test positivity rate
TSI: Temperature suitability index
WHO: World Health Organization
