# Supplementary material for: Spatio-temporal modelling of routine health facility data for malaria risk micro-stratification in mainland Tanzania
Source: Sci Rep. 2023 Jun 30;13:10600. doi: 10.1038/s41598-023-37669-x (PMC10313820; doi:10.1038/s41598-023-37669-x)
Supplement: Supplementary file 1 — Supplementary Information. [file 41598_2023_37669_MOESM1_ESM.docx]

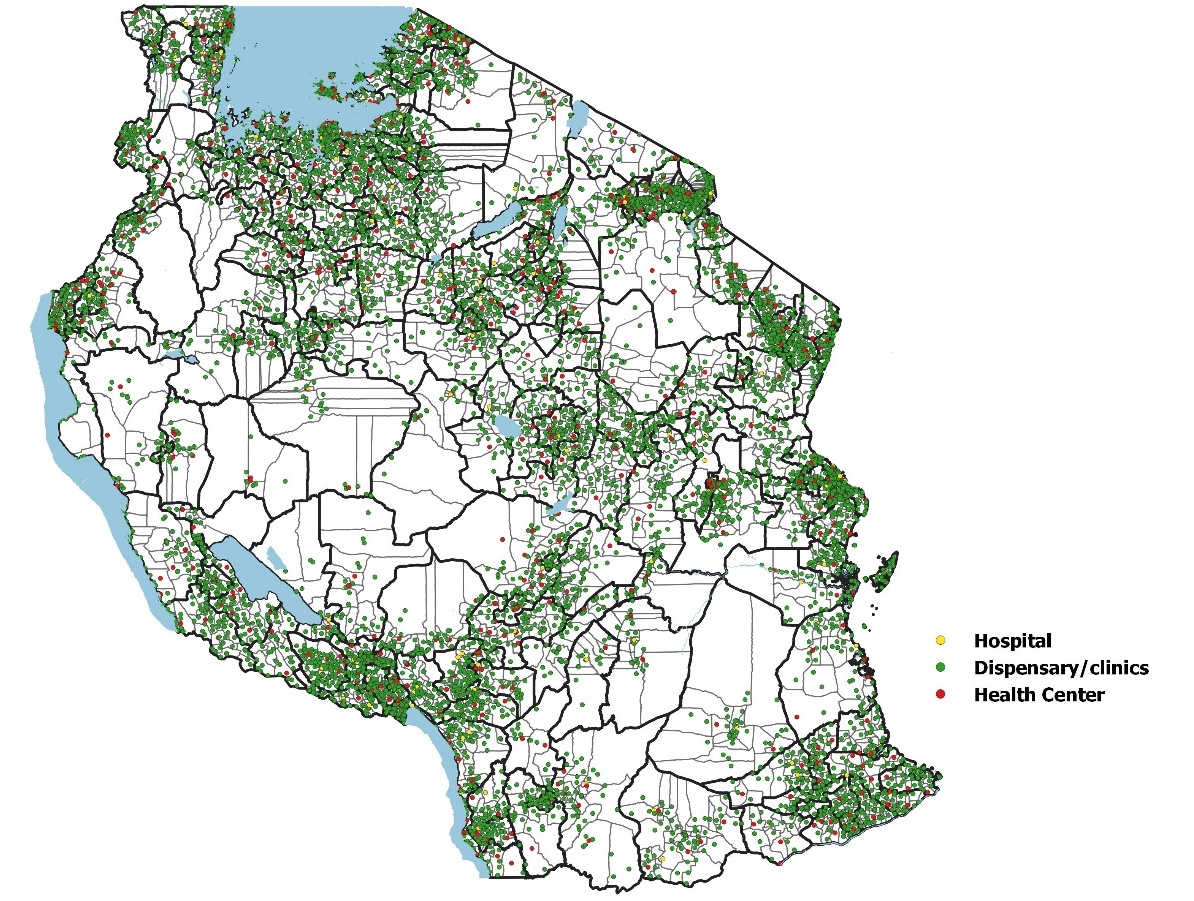


**Figure S1**: Location of health facilities (HFs) by type (n=7,878)*

*For 157 (2%) of the total HFs, the ward name in the master HF list did not appear in the existing ward shape file and therefore the geo-coordinate was used to guide the ward location in the shape file. The geo coordinates for another 180 (2%) HFs could not be obtained from the master HF list and thus open source platforms such as Google Earth was used to retrieve the information. The geo coordinates for 671 (8.5%) HFs did not match the ward name indicated in the master HF list and therefore changed to reflect the correct ward.

**Text S1: Covariate Selection**

*Covariates*

The following covariates known to influence malaria transmission were considered for model selection (Figure S2) and were extracted per ward polygon in R software.

- *Digital Elevation Model (DEM):* DEM data were obtained from Regional Centre for Mapping of Resources for Development and available at 30-meter resolution (https://opendata.rcmrd.org/datasets/rcmrd::tanzania-srtm-dem-30-meters/about). It is a representation of the topographic surface of the Earth.
- *Precipitation*: Precipitation data for 2017-2019 was obtained from the Climate Hazards Group InfraRed Precipitation with Stations (CHIRPS Version 2.0) (https://data.chc.ucsb.edu/products/CHIRPS-2.0/). CHIRPS-2.0 is an open source platform with time series data available at 0.05^o^ x 0.05^o^ spatial resolution and produced by combining quasi-global satellite and observation based precipitation estimates.
- *Enhanced Vegetation Index (EVI):* EVI data for 2017-2019 were obtained from Moderate-resolution Imaging Spectroradiometer (MODIS) sensor imagery (http://modis.gsfc.nasa.gov/data/). This indicator is a measure of photosynthetic activity and widely used for monitoring vegetation conditions.
- *Temperature Suitability Index (TSI*): TSI is a representation of the optimal development of P. falciparum sporozoite and reflects the transmission suitability. It was developed in 2011 at 1 × 1 km spatial resolution [Gething et al. 2011].
- *Average Health Facility Reporting Rates*: The completeness in submission of malaria reports varies across the facilities of mainland Tanzania. To account for the differing rates, the average facility reporting rate was computed per year and per ward.
- *Nighttime lights (NTL)*: This indicator was used to represent the level of urbanization and as a proxy for socioeconomic status [Zhao et al. 2019]. The data were derived from DMSP-OLS (2000-2013) and Visible Infrared Imaging Radiometer Suite (VIIRS) (from 2013 - 2020) onboard the Suomi National Polar Partnership (NPP) satellite launched in 2011 with a spatial resolution of approximately 1 km. The data contain the mean of visible band digital number values of cloud-free light detections (<https://www.nasa.gov/nex/data>).
- *Humidity:* This indicator is a measure of the amount of water vapor in the atmosphere. Satellite water vapor estimates were obtained from Moderate-resolution Imaging Spectroradiometer (MODIS) sensor imagery (<https://neo.gsfc.nasa.gov/>) at 5x5 1-km pixel resolution. The amount of water vapor affects the longevity of the malaria vector thereby enabling the full development of the parasites in areas with high humidity and thus transmission.


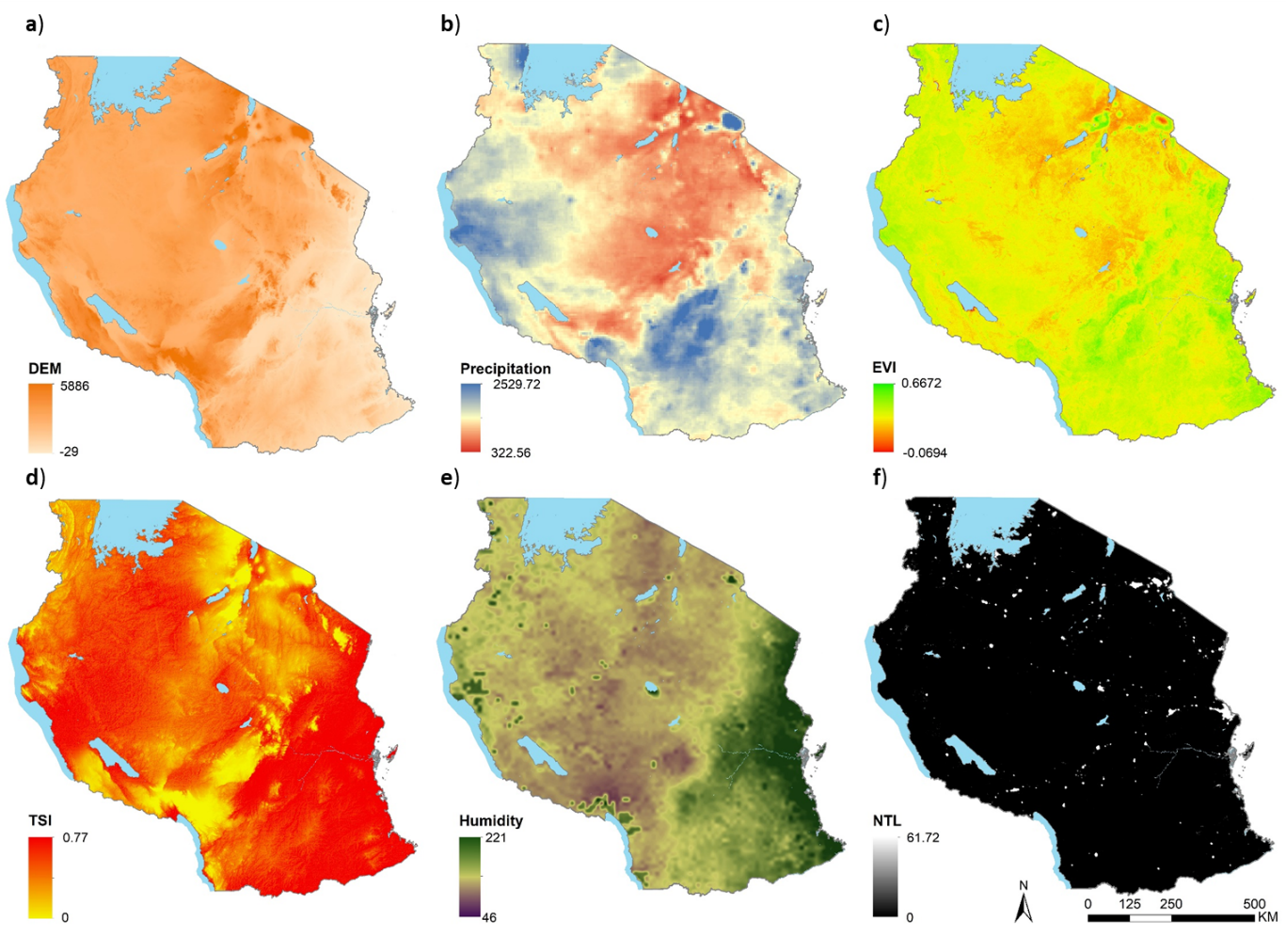


**
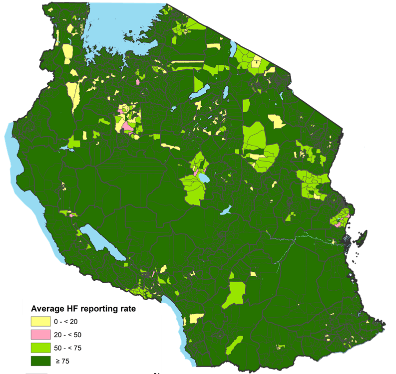
g**)

**Figure S2:** Maps of covariates showing: a) Digital Elevation Model (DEM); b) Precipitation; c) Annual mean enhanced vegetation index (EVI); d) Temperature Suitability Index (TSI); e) Humidity; f) Night-time lights (NTL); g) Annual mean health facility reporting rates

*Covariate selection process*

In order to select the minimum set of covariates for the model, a statistical analysis was performed using the leap algorithm available under the *bestglm* package in R. A cross- validation (CV) approach was implemented based on a ten-fold CV method and the model with the best CV score was selected. The covariates selected from this procedure included DEM, NTL, TSI and EVI. Figure S3 shows the decay in CV error based on the subset models. However, since DEM and TSI showed high collinearity, only TSI was retained. The rationale being that temperature is a key determinant of environmental suitability for malaria transmission [Gething et al. 2011] and this index incorporates the mechanism of temperature dependency within the malaria transmission cycle.


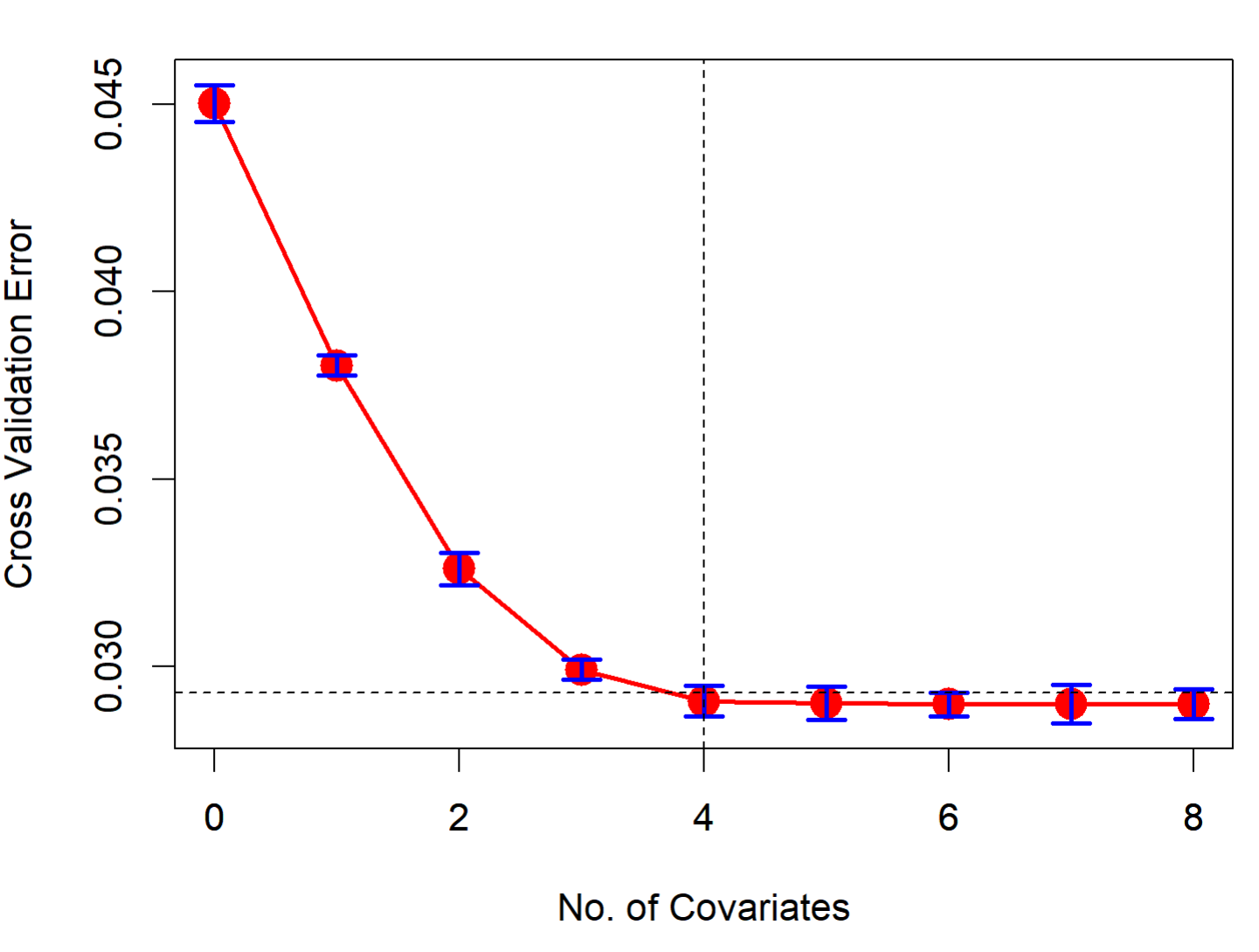


**Figure S3:** Model selection with estimated cross-validation error in red across the number of covariates using 10-fold cross-validation method

**Text S2: Model Specifications**

*Model description*

The Besag-York-Mollié 2 Model (BYM2) developed takes into account that data may be spatially correlated and observations in neighboring adjacent wards may be more similar than observations in wards that are farther away. It includes a spatial random effect that is assigned a CAR distribution and smoothens the data according to a neighborhood structure, and an unstructured exchangeable component that models uncorrelated noise. The BYM2 model allows to simultaneously capture the heterogeneity and clustering of TPR at ward levels [Idrissu et al. 2018].

The selected covariates from a preliminary analysis, total mRDT confirmed cases and the total tested for malaria were used to model the spatial and temporal variation of TPR and provide posterior predictions at unsampled locations with associated uncertainty. The selection of other prior information of the parameters followed the standard fixed prior specifications [Illian et al. 2013]. R-INLA performs approximate Bayesian inference for the class of latent Gaussian models using analytical approximation and numerical algorithms [Blangiardo et al. 2015].

Exceedance probability (EP) and non-exceedance probabilities (NEP) were used to quantify the uncertainty in the likelihood of estimates of TPR to be above or below the pre-defined policy relevant thresholds respectively. For instance, the probability that the risk of an area is higher than a value c is expressed as $P(pi>c)$. The probability was thus calculated by using the formula $P\left( pi>c \right)=1- P\left( pi\leq c \right)$. Values of the probabilities close to 100% indicate that the $P\left( pi>c \right)$ is highly likely to be above the threshold whilst those close to 0% are highly likely to be below the threshold. Values close to 50% indicate high levels of uncertainty. For TPR, a threshold of ≥30% was used to represent the wards with high risk whilst a threshold of <5% was used to represent the wards with very low malaria risk.

*Neighborhood matrices*

Figure S4 shows the adjacency matrices created for mainland Tanzania. Wards sharing a common boundary were considered neighboring wards. The strength in the estimates from these wards were borrowed in time and space for predicting the TPR estimates.


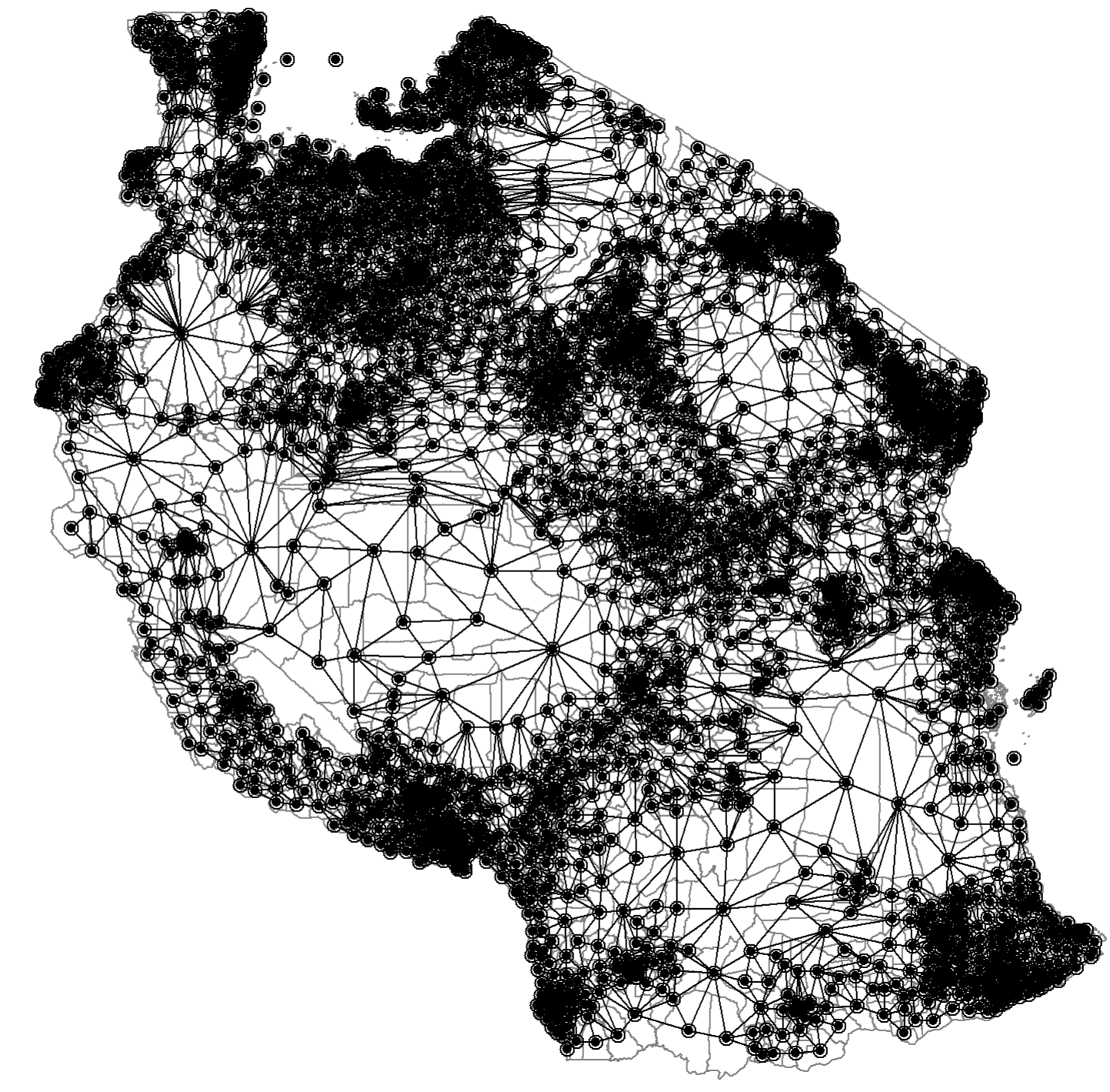


**Figure S4**: Spatial neighborhood matrices for the wards of mainland Tanzania

*Model Selection and Validation Plots*

In order to test the goodness of fit, CAR models with different specifications of the spatio-temporal structures were implemented (Table S1). The model performance was validated by computing the MAE, RMSE and R2 on the 10% test hold-out dataset.

**Table S1:** Different specifications of CAR model to test goodness of fit

| **Model description** | **Specification** | **DIC** | **R2** | **MAE** | **RMSE** |
| --- | --- | --- | --- | --- | --- |
| A. Without spatial random effect | $logit\left( P\left( j,k \right) \right)=\beta_{0}+X\left( j,k \right)^{'}\beta+v_{j}$ | 306,978.1 | 0.91 | 0.04 | 0.06 |
| B. With spatial random effect | $logit\left( P\left( j,k \right) \right)=\beta_{0}+X\left( j,k \right)^{'}\beta+v_{j}+u_{j}$ | 307,065.9 | 0.91 | 0.04 | 0.06 |
| C. With spatial and temporal random effect | $logit\left( P\left( j,k \right) \right)=\beta_{0}+X\left( j,k \right)^{'}\beta+v_{j}+u_{j}+\gamma_{j}$ | 304,069.5 | 0.91 | 0.04 | 0.06 |

$\beta_{0}$ the intercept; $X(j,k)$ is a set of selected covariates; $\beta$ are the corresponding regression parameters; $u_{j}$ corresponds to the CAR structured spatial random effect that smoothens the data according to a neighbourhood structure;$v_{j}$corresponds to the unstructured exchangeable component using independent and identically distributed (i.i.d) random effect and $\gamma_{k}$ is the temporal random effect specified using i.i.d zero-mean normally distributed random effect.

The semi-variogram of the residuals of the selected model C showed minimum spatial autocorrelation after modelling suggesting that the spatial structure in the data was accounted for (Figure S5).

1.
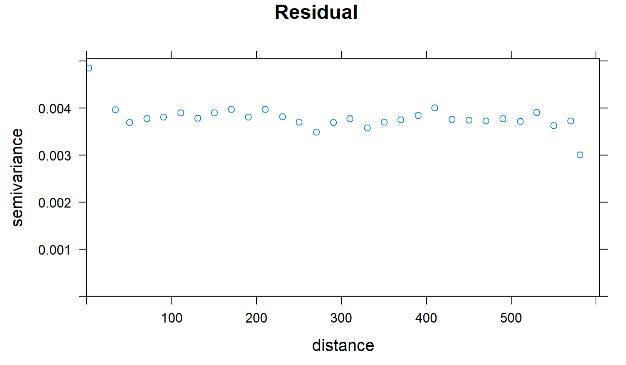
 *B.*

*
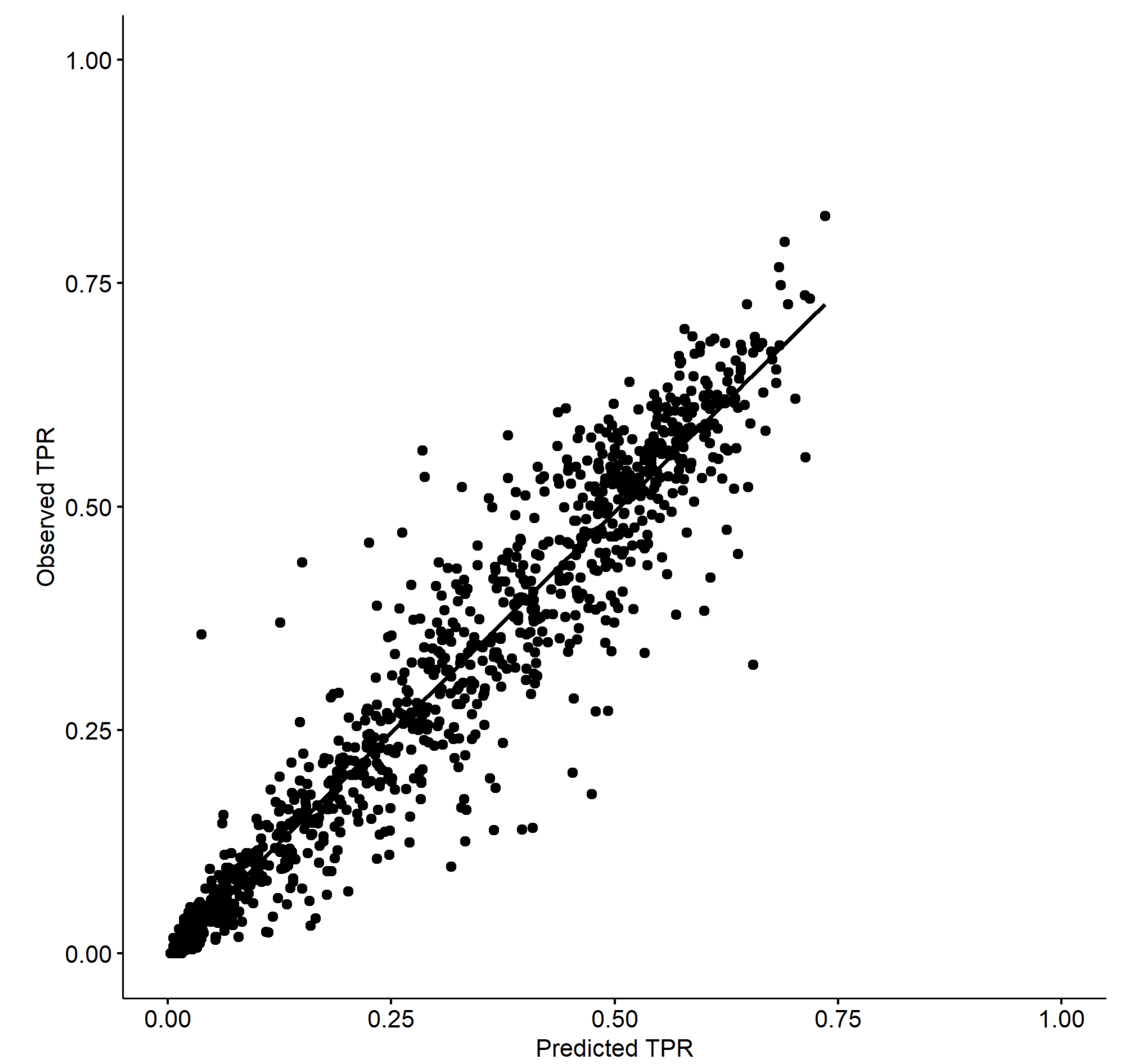
*

**Figure S5**: A) The scatter plot of observed TPR against predicted modelled TPR for the 10% test dataset. B) Semi-variogram of model residuals with minimum spatial structure of the selected model C


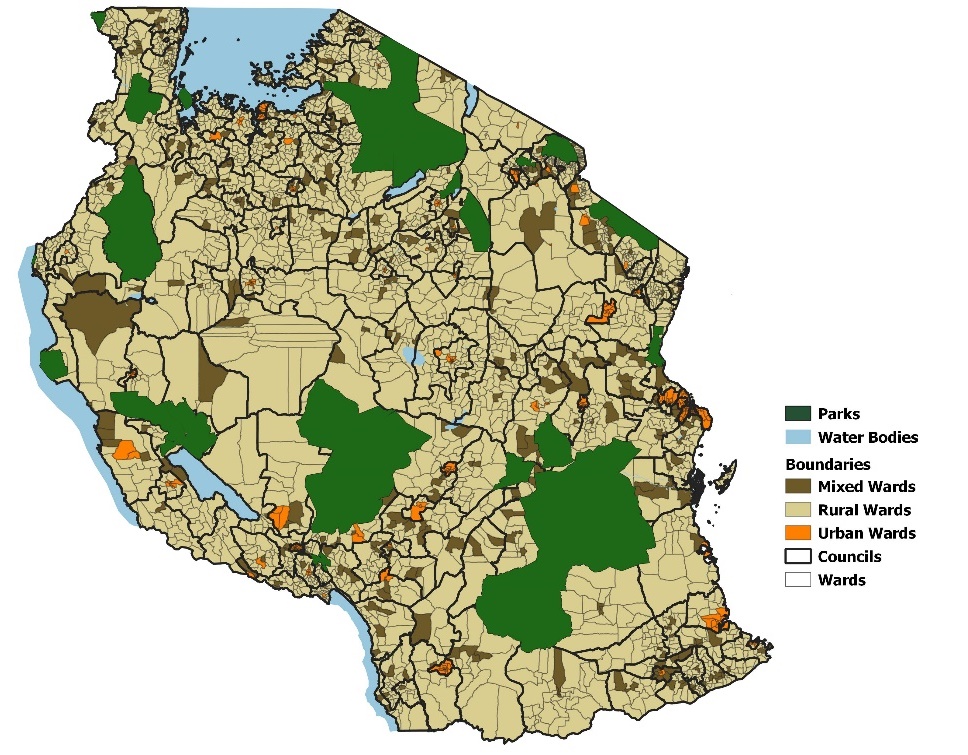


**Figure S6**: Administrative boundaries and distribution of urban (n=2,427), rural (n=514) and mixed wards (n=370) in mainland Tanzania.

**Table S2:** Selected routine indicator cut-offs to categorize into risk groups

| **Prevalence in School Children (*Pf*PR_5-16_)** | **Very Low risk (*Pf*PR_5-16_ <1%)** | **Low risk**  **(*Pf*PR_5-16_ 1-<5%)** | **Moderate risk**  **(*Pf*PR_5-16_ 5-<30%)** | **High risk**  **(*Pf*PR_5-16_ ≥30%)** |
| --- | --- | --- | --- | --- |
| 1. mRDT Test Positivity Rate (TPR) | <5 | 5-<15 | 15-<30 | ≥30 |
| 2. Annual Parasite Incidence (API) | <10 | 10-<50 | 50-<120 | ≥120 |
| 3. Test Positivity Rate in Pregnant Women (ANC TPR) | <0.8 | 0.8-<3 | 3-<8 | ≥8 |

**References**

Blangiardo M. & Cameletti M. (2015). Spatial and Spatio-temporal Bayesian Models with R-INLA. Chichester: Wiley.

Funk C, Peterson P, Landsfeld M, Pedreros D, Verdin J, Shukla S, et al. The climate hazards infrared precipitation with stations—a new environmental record for monitoring extremes. Scientific Data. 2015;2(1):150066. doi: 10.1038/sdata.2015.66

Gething PW, Van Boeckel TP, Smith DL, Guerra CA, Patil AP, Snow RW, et al. Modelling the global constraints of temperature on transmission of Plasmodium falciparum and P. vivax. Parasites & vectors. 2011;4(1):1-11

Iddrisu, A.-K., Alhassan, A. and Amidu, N. (2018) Investigating Spatio-Temporal Pattern of Relative Risk of Tuberculosis in Kenya Using Bayesian Hierarchical Approaches. Journal of Tuberculosis Research, 6, 175-197

Illian J. B., Sørbye S. H. & Rue H. (2012). A toolbox for fitting complex spatial point process models using integrated nested Laplace approximation (INLA). Ann. Appl. Statist. 6, 1499–530.

Zhao M, Zhou Y, Li X, Cao W, He C, Yu B, et al. Applications of Satellite Remote Sensing of Nighttime Light Observations: Advances, Challenges, and Perspectives. Remote Sensing. 2019;11(17):1971. PubMed PMID: doi:10.3390/rs11171971
